# Effects of dietary *Kleinhovia hospita* and *Leucaena leucocephala* leaves on rumen fermentation and microbial population in goats fed treated rice straw

**DOI:** 10.1007/s11250-017-1388-3

**Published:** 2017-08-28

**Authors:** Muideen Adewale Ahmed, Kazeem Dauda Adeyemi, Mohamed Faseleh Jahromi, Shokri Jusoh, Abdul Razak Alimon, Anjas Asmara Samsudin

**Affiliations:** 1 Department of Animal Science, Faculty of Agriculture, Universiti Putra Malaysia, UPM, 43400 Serdang, Selangor Darul Ehsan Malaysia; 2 Animal Production Laboratory, Institute of Tropical Agriculture, Universiti Putra Malaysia, Serdang, Selangor Malaysia

**Keywords:** *Kleinhovia hospita*, *Leucaena leucocephala*, Microbial population, Rumen fermentation

## Abstract

The effects of partial replacement of dietary protein by forages on rumen fermentation and microbiology in goats were examined. Four fistulated Boer bucks were used in a 4 × 4 Latin square design. The goats were fed 60% of urea-treated rice straw and 40% dietary treatment (*Kleinhovia hospita* (KH), *Leucaena leucocephala* (LL), mixture of *K. hospita* with *L. leucocephala* (KHLL)) and concentrate as the control. Rumen fluid from the animals was collected at 0, 2, 4, 6, and 12 h postprandial for analysis. The KHLL diet had a greater (*P* < 0.05) molar proportion of acetate than the control diet throughout the sampling period. At 6 h postprandial, the KHLL goats had a significantly lower (*P* < 0.05) ammonia nitrogen than the goats fed other diets. The molar proportion of propionate (24.7 and 25.8 mol/100 mol) was greater in the rumen of KHLL goats compared with those fed other diets at 2 and 12 h postprandial, respectively. The KHLL diet had lower (*P* < 0.05) butyrate than other dietary treatments. At 4 h postprandial, the control goats had a lower (*P* < 0.05) population of total bacteria while the KHLL goats had a greater (*P* < 0.05) population at 4 and 12 h postprandial compared with those fed other diets. The LL, KH, and KHLL goats had lower (*P* < 0.05) populations of protozoa and methanogens and a greater (*P* < 0.05) population of *Ruminococcus albus* compared with the control goats. The KHLL leaves could be fed to goats without compromising rumen metabolism.

## Introduction

One of the major constraints to sustainable ruminant production particularly in the tropics is the lack of affordable and available feed resources (Devendra and Leng 2011). This is due to the high cost of conventional feedstuffs and the competition between man and livestock industry for the available conventional feedstuffs (Leng 2008). Thus, the use of cheaper feed alternatives with high proximity and year-round availability has been the subject of research in recent years.

Various tree forages have been used as supplement of protein source in the diets of ruminants. Nonetheless, the presence of plant secondary metabolites in the tree forages could limit their nutritional value (Kim et al. 2015). In addition, the effects of tree forages on rumen metabolism and growth performance in ruminants have been highly inconsistent and variable in the published literature. This development has stimulated the interest for additional studies in different production systems to permit tailored decisions and informed choices in the utilization of tree forages in ruminant nutrition.

To the best of our knowledge, the efficacy of *Kleinhovia hospita* as ruminant feed has not been investigated. Therefore, the objective of this study was to determine the effect of feeding *K. hospita* (KH), *Leucaena leucocephala* (LL), and *K. hospita* mixed with *L. leucocephala* (KHLL) on rumen fermentation profile and microbial population in goats fed with urea-treated rice straw.

## Materials and methods

### Animal welfare and ethics

This study was carried out following the guidelines of the research policy of the Universiti Putra Malaysia (UPM) on animal welfare and ethics. The care of the experimental goats was in accordance with Malaysian standards.

### Experimental animals and feeding

The experiment was carried out at Field 2 (longitude 101°42′09.4″E and latitude 3°00′27.7″N) Ruminant Farm, Department of Animal Science, Faculty of Agriculture, UPM. Four crossbred Boer bucks weighing 30–32 kg and fitted with permanent ruminal cannulas were used in a 4 × 4 Latin square design. Each goat was housed separately and fed 60% treated rice straw (basal diet) and 40% dietary treatment based on 3% body weight. The dietary treatments were T1: concentrate supplement (control), T2: *K. hospita* leaves, T3: *L. leucocephala*, and T4: mixture of *K. hospita* and *L. leucocephala*. The soybean meal and palm kernel cake were partially or wholly replaced by the forages. The adaptation period was 2 weeks prior to the commencement of the experiment and the goats were fed once a day (0800 h). A 5-day interval of non-treatment diet was allowed to avoid residual effect of the dietary treatments. The goats had ad libitum access to water throughout the trial. The rumen samples were collected at 0, 2, 4, 6, and 12 h postprandial. The pH of the rumen fluid was measured immediately after collection using a pH meter (Mettler-Toledo Ltd., England). The samples were squeezed through four layers of cheesecloth and frozen (− 20 °C) until further analysis.

### Feed analysis

The proximate compositions of the treatments were presented in Table 1 and  determined according to the protocol of AOAC (2007) while the detergent fibers were determined according to the method of Van Soest et al. (1991). The gross energy content of the treatments was determined using adiabatic bomb calorimeter (Leco Corporation, MI 49085, USA). Estimation of total polyphenol and tannins was done following the procedure of Makkar et al. (1993).

**Table 1:** Chemical composition of the experimental diets

Ingredients (%)	CD	KH	LL	KHLL	RS
Treated rice straw (%) from the total ration	60.00	60.00	60.00	60.00	
Corn	19.30	19.30	19.30	19.30	
Palm kernel cake	57.80	15.30	27.30	–	
Soybean meal	20.90	13.50	13.50	–	
KH	–	50.00	–	20.00	
LL	–	–	38.00	58.74	
CaCO_3_	0.50	0.50	0.50	0.50	
NaCl	0.50	0.50	0.50	0.50	
Min. premix	1.00	1.00	1.00	1.00	
Chemical composition % DM					
Dry matter	90.50	91.00	90.40	92.00	96.60
Ash	6.08	7.01	6.90	7.84	12.90
Organic matter	93.90	93.00	93.00	92.20	87.10
Crude protein	20.00	19.80	19.90	19.70	5.00
Ether extract	1.39	2.39	1.48	3.00	1.63
Crude fiber	9.61	10.30	13.00	13.70	36.30
Neutral detergent fiber	49.60	50.80	43.50	39.20	80.80
Acid detergent fiber	19.40	18.80	18.60	19.40	48.60
Lignin	8.00	10.50	13.00	13.30	31.60
Gross energy MJ/kgDM	14.50	15.40	15.50	15.90	14.80
Tannin (%)	–	1.26	0.95	1.39	–

CD (control diet), KH (*Kleinhovia hospita* diet), LL (*Leucaena leucocephala* diet), KHLL (*Kleinhovia hospita* and *Leucaena leucocephala* mixed diet), RS (rice straw)

### Estimation of rumen fermentation parameters

The determination of the VFA concentration and molar proportions were achieved by gas chromatography (Agilent 69890N Series) fitted with a flame ionization detector. About three to five drops of 10% H_2_SO_4_ was added to 5 mL of rumen fluid and kept at − 20 °C until further analysis Cottyn and Boucque 1968 using 0.5 mL of 20 mmol/L 4-methyl-*n*-valeric acid as the internal standard. The NH_3_-N concentration of the rumen fluid was evaluated as described by Parsons et al. (1984). The absorbance was determined at 640 nm using a Spectro SC spectrophotometer (Labomed Inc., Culver City, CA).

### Microbial quantification

#### Rumen microbial DNA extraction

The extraction of DNA in rumen fluid DNA was determined by using the cetyltrimethylammonium bromide (CTAB) extraction procedure as described by Samsudin et al. (2011). The extracted DNA pellet was resuspended in 50 μL TE and stored at − 20 °C until further analysis. BioPhotometer plus (Eppendorf W/Hellma Traycell 952000070) was used to determine the extracted DNA concentration.

#### Quantitative real-time PCR

Species-specific quantitative real-time PCR was carried out using CFX96 Touch Real-Time PCR Detection System (BioRad, USA) with an optical grade plate of SYBR Green mix detection. The reaction of the PCR was executed on a total volume of 25 μL using QuantiFast SYBR® Green RT-PCR Kit. The reaction of each sample was made up of 12.5 μL SYBR Green Supermix, 1 μL of forward primer, 1 μL of reverse primer, 2 μL of DNA samples, and 9.5 μL DNA template and RNAse free water. The mixed samples were placed in Axygen scientific 0.2 RT PCR strips. The PCR reaction conditions applied to each well was as follows: 5-min initial incubation at 94 °C then 40 cycles at 94 °C for 20 s for denaturation followed by annealing temperature (depending on the primers) (Table 2) for 30 s, and extended to 72 °C for 20 s (Jahromi et al. 2013).

**Table 2:** Primers for real-time PCR assay (Jahromi et al. 2013)

Target group	Sequence 5′–3′	Product size (bp)	Annealing temperature (°C)
Total bacteria F	CGGCAACGAGCGCAACCC	145	55
Total bacteria R	CCATTGTAGCACGTGTGTAGCC		
Methanogens (mcrA)-F	TTCGGTGGATCDCARAGRGC	140	58
Methanogens (mcrA)-R	GBARGTCGWAWCCGTAGAATCC		
*Ruminococcus albus* F	CCCTAAAAGCAGTCTTAGTTCG	175	55
*Ruminococcus albus* R	CCTCCTTGCGGTTAGAACA		
*Ruminococcus flavefaciens* F	TCTGGAAACGGATGGTA	259	60
*Ruminococcus flavefaciens* R	CCTTTAAGACAGGAGTTTACAA		
*Fibrobacter succinogenes* F	GTTCGGAATTACTGGGCGTAAA	122	55
*Fibrobacter succinogenes* R	CGCCTGCCCCTGAACTATC		
Protozoa F	CTTGCCCTCYAATCGTWCT	223	55
Protozoa R	GCTTTCGWTGGTAGTGTATT		

Primers used to quantify the population of different groups of microorganisms are shown in Table 2. The number of copies of a template DNA per milliliter of elution buffer was quantified using the formula that is available online. (http://www.uri.edu/research/gsc/resources/cndna.htmL):

$$ \mathrm{Number}\kern0.17em \mathrm{of}\kern0.17em \mathrm{copies}=\frac{\mathrm{Amount}\kern0.17em \mathrm{of}\;\mathrm{DNA}\left(\upmu \mathrm{g}/\mathrm{mL}\right)\times 6.022\times {10}^{23}}{\mathrm{Length}\kern0.17em \mathrm{of}\;\left(\mathrm{bp}\right)\times {10}^9\times 650}\times 100 $$

$$ \mathrm{The}\  \mathrm{amplification}\  \mathrm{efficiency}\  \mathrm{was}\  \mathrm{estimated}\  \mathrm{using}\  \mathrm{the}\  \mathrm{equation}\ \mathrm{E}=\left({10}^{\hbox{--} 1/\mathrm{slope}}-1\right)\times 100\%. $$

#### Statistical analysis

Data obtained for all variables were analyzed using the MIXED procedure of SAS (2012). Diet, time, and interaction between diet and time were fitted as a fixed effect in a repeated measure analysis. Differences between means were separated by the Duncan multiple range test at *P* < 0.05.

## Results and discussion

### Rumen fermentation profile

#### Rumen pH

The mean ruminal pH of goats fed different dietary supplements is presented in Table 3. The mean pH ranged from 6.16 to 7.05 and was within the range for normal pH for optimum rumen metabolism (Valente et al. 2016). Dietary treatments and sampling time had significant effects (*P* < 0.05) on rumen pH in goats. The pH value decreased over sampling time. This observation suggests an increase in the rumen fermentation of the dietary treatments. The KHLL diet had a greater pH than the control diet throughout the sampling period perhaps due to the presence of polyphenol (Kim et al. 2015). A similar trend was observed for KH and LL diets though the results were inconsistent. The lower rumen pH in the KHLL goats could be due to the lower total VFA (Table 4) induced by the greater amount of polyphenols (% tannin) in the diets (Table 1). Changes in rumen pH of goats fed a different diet with respect to sampling time are an indication of varying buffering capacity and degradation of feed in the rumen (Castillo-González et al. 2014).

**Table 3:** Mean ruminal pH and ammonia nitrogen in goats as influenced by dietary treatments and sampling time

Parameter	Time (h)						SEM	*P* value	DXT
	Diet	0	2	4	6	12			
pH	CD	6.79^bw^	6.69^cw^	6.61^cwx^	6.47^cy^	6.16^cy^	0.238	0.0001	
	KH	6.95^abw^	6.81^bx^	6.69^bcy^	6.60^by^	6.33^bcz^	0.236	< 0.0001	0.0012
	LL	6.93^abw^	6.81^bwx^	6.75^abx^	6.58^bcy^	6.41^bz^	0.203	< 0.0001	
	KHLL	7.05^aw^	6.90^ax^	6.86^ax^	6.75^ay^	6.67^az^	0.145	< 0.0001	
	*P* value	0.036	0.002	0.008	0.006	0.0008			
Ammonia nitrogen (%)	CD	12.10^x^	21.60^wx^	23.30^aw^	15.60^wx^	0.95^y^	8.93	0.0100	
	KH	7.38^y^	14.90^x^	21.80^aw^	11.70^xy^	0.72^z^	8.15	0.0007	
	LL	6.55^y^	14.90^x^	22.60^aw^	14.30^x^	0.85^z^	8.36	0.0005	0.214
	KHLL	4.25^y^	12.90^x^	18.20^bw^	12.50^x^	0.31^z^	7.61	< 0.0001	
	*P* value	0.101	0.075	0.011	0.683	0.055			

CD (control diet), KH (*Kleinhovia hospita* diet), LL (*Leucaena leucocephala* diet), KHLL (*Kleinhovia hospita* and *Leucaena leucocephala* mixed diet), SEM (standard error of mean), DXT (diet-time interaction for all the treatments)

^abcd^Means with different superscripts along the same column are significantly different (*P* < 0.05)

^wxyz^Means with different superscripts along the row are significantly different (P < 0.05)

**Table 4:** Total and molar proportions of volatile fatty acids in the rumen of goats as influenced by dietary treatments and sampling time

	Time (h)						SEM *P* value DXT		
	Diet	0	2	4	6	12			
Total VFA (mM)	CD	93.8	104^b^	80.5^b^	83.7	104	11.0	0.07	
	KH	91.2^x^	88.0^dx^	86.3^bx^	89.9^x^	111^w^	10.2	0.05	
	LL	100.0	111.0^a^	104.0^a^	104.0	99.6	4.28	0.7	0.09
	KHLL	86.4^xy^	95.8^cwx^	79.4^cy^	87.9^xy^	103^w^	9.25	0.04	
	*P* value	0.57	0.002	0.02	0.1	0.63			
Acetate (mol/100 mol)	CD	55.7^b^	55.3^b^	53.7^b^	54.1^c^	53.7^b^	0.96	0.29	
	KH	56.5^b^	57.1^b^	58.3^a^	56.4^b^	56.5^ab^	0.8	0.16	
	LL	54.4^b^	57.4^b^	57.5^ab^	56.7^b^	53.8^b^	1.74	0.23	0.2071
	KHLL	60.1^a^	60.5^a^	61.2^a^	60.7^a^	59.7^a^	0.56	0.8	
	*P* value	0.02	0.04	0.05	0.004	0.03			
Propionate (mol/100 mol)	CD	20.2^x^	23.4^bw^	22.7^w^	22.6^w^	22.8^bw^	1.38	0.002	
	KH	20.9	21.8^c^	22.0	22.3	23.9^b^	0.61	0.17	
	LL	20.6	23.4^b^	23.1	22.5	23.8^b^	1.11	0.25	0.14
	KHLL	22.3^x^	24.7^awx^	23.9^wx^	24.1^wx^	25.8^aw^	1.28	0.04	
	*P* value	0.32	0.01	0.19	0.14	0.01			
Butyrate (mol/100 mol)	CD	14.2^y^	14.1^by^	15.9^ax^	16.7^bwx^	18.3^aw^	1.78	0.01	
	KH	15.2^z^	15.9^ayz^	16.9^axy^	17.9^awx^	18.6^aw^	1.41	0.01	
	LL	16.9^wx^	13.1^by^	14.6^axy^	16.2^bwxy^	18.9^aw^	2.23	0.04	0.002
	KHLL	12.7^wx^	10.7^cy^	11.9^bx^	13.3^cw^	13.4^bw^	1.12	0.002	
	*P* value	0.12	0.002	0.02	0.0002	0.005			

CD (control diet), KH (*Kleinhovia hospita* diet), LL (*Leucaena leucocephala* diet), KHLL (*Kleinhovia hospita* and *Leucaena leucocephala* mixed diet), SEM (standard error of mean), DXT (diet time interaction for all the treatments)

^abcd^Means with different superscripts along the same column are significantly different (*P* < 0.05)

^wxyz^Means with different superscripts along the row are significantly different (*P* < 0.05)

#### Rumen ammonia N

Ammonia nitrogen serves as an effective indicator of protein degradation in the rumen and it is the major source of microbial protein synthesis in the rumen (Adeyemi et al. 2016). The concentration of ruminal ammonia nitrogen in goats as influenced by dietary treatments and sampling time is presented in Table 3. Sampling time had a significant impact (*P* < 0.05) on the concentration of ammonia nitrogen in goats. Regardless of the dietary treatment, the concentration of ammonia nitrogen increased from 0 to 4 h postprandial and declined afterwards. This implies that at 4 h postprandial, the proteolytic activities of the microflora were optimum.

The range and trend of ammonia concentration over time are in tandem with the findings of Yanez-Ruiz et al. (2004). However, at 6 h postprandial, the KHLL goats had a significantly lower (*P* < 0.05) ammonia nitrogen than goats fed other diets. This observation could be attributed to the presence of greater amount of phenolic compound (tannin) in the KHLL diet compared with other diets. The reduction in the ruminal degradation of protein associated with the reduction in ruminal ammonia nitrogen is the most common effect of phenolic compounds (Pitta et al. 2010). The low rumen ammonia–N concentration in KHLL goats could be due to reduced recycling of bacterial protein owing to a lower protozoa population (Abubakr et al. 2013).

#### Total and molar proportions of VFA

The total and molar proportions of VFA in the rumen of goats fed different dietary treatments are presented in Table 4. Dietary treatments had no effect (*P* > 0.05) on the concentration of ruminal total VFA in goats at 0, 6, and 12 h postprandial. At 4 and 6 h postprandial, the total VFA varied among the treatments. This observation could be due to the variation in the concentration, types, and sources of tannin in the diets. Sampling time influenced the concentration of total VFA in KHLL and KH goats but the changes were inconsistent. Similar inconsistencies in total VFA were observed in goats fed leaf meal-based diets (Singh et al. 2011).

Dietary treatments had a significant impact on the molar proportion of acetate in the rumen of goats. The KHLL goats had greater (*P* < 0.05) concentrations of acetate than the control goats throughout the sampling period. The molar proportion of acetate did not differ between the control goats and those fed the LL and KH diets at 0, 2, 4, and 12 h postprandial. Sampling time did not affect (*P* > 0.05) the molar proportion of acetate in goats. The greater acetate in the rumen of KHLL goats could be due to the higher cellulose content in the diets resulting to greater feed degradation. The presence of acetate in ruminant is essential as it increases colonic blood flow and enhances ileal motility and milk fat formation (Scheppach 1994). The molar proportion of propionate was greater (*P* < 0.05) in the rumen of KHLL goats compared with those fed other diets at 2 and 12 h postprandial. The higher proportion of propionate shows better utilization of the dietary energy since the formation of propionate reduces the amount of hydrogen gas available for the synthesis of methane. The KHLL diet had lower (*P* < 0.05) butyrate than other dietary treatments. This observation could be attributed to the reduced population of protozoa in the rumen of KHLL goats. A decrease in butyrate and an increase in propionate often characterize the absence of protozoa (Kara et al. 2017). The current observation is consistent with the findings of Franzolin et al. (2010) who observed a lower butyrate concentration in the rumen of cattle and buffalo fed citrus pulp and soybean meal.

### Rumen microbial profile

#### Total bacteria

The rumen microbiota in goats fed different dietary treatments and sampled over different postprandial periods is shown in Table 5. Dietary treatments had no effect (*P* < 0.05) on the population of total rumen bacteria in goats at 0 h postprandial. A similar finding was observed in goats fed pakar leaves and green oats (Singh et al. 2011). At 4 h postprandial, the control goats had a lower (*P* < 0.05) population of total bacteria while the KHLL goats had greater (*P* < 0.05) populations at 4 and 12 h postprandial compared with those fed other diets. The greater total bacteria in goats fed tree forages could be attributed to the lower population of protozoa, which caused less engulfment of the bacteria. Postprandial sampling time influenced the population of total bacteria in goats fed the control, LL, and KH diets. However, the changes were inconsistent.

**Table 5:** Rumen microflora (Log_10_ copy no/mL) in goats as influenced by dietary treatments and sampling time

	Diet	Time (h)			SEM	*P* value	DXT
		0	4	12			
Total bacteria	CD	9.37^x^	5.49^cz^	8.77^by^	2.090	0.0002	0.0371
	KH	9.02^x^	8.84^bx^	8.42^cy^	0.308	0.0069	
	LL	9.09^y^	10.21^ax^	8.77^by^	0.755	0.0026	
	KHLL	9.33	9.52^ab^	9.23^a^	0.150	0.8550	
	*P* value	0.72	0.0004	0.0016			
Total protozoa	CD	3.98^y^	6.94^ax^	3.39^y^	1.900	0.010	
	KH	4.27^x^	3.40^bxy^	3.30^y^	0.534	0.055	0.021
	LL	4.02	4.15^ab^	3.37	1.450	0.188	
	KHLL	3.72^xy^	4.00^abx^	2.83^y^	0.623	0.054	
	*P* value	0.433	0.052	0.453			
Methanogen archaea	CD	6.94^y^	8.39^ax^	7.01^ay^	0.820	0.010	0.001
	KH	6.92	6.70^c^	6.39^b^	0.930	0.586	
	LL	6.79	7.47^b^	7.00^a^	0.344	0.173	
	KHLL	6.79^x^	6.49^cx^	6.00^by^	0.396	0.011	
	*P* value	0.906	0.002	0.014			
*Ruminococcus albus*	CD	6.19^x^	3.33^by^	5.44^cx^	1.480	0.035	0.0037
	KH	5.39^y^	5.19^ay^	6.02^bx^	0.432	0.024	
	LL	5.82	5.95^a^	6.33^a^	0.267	0.191	
	KHLL	6.02	6.05^a^	6.29^a^	0.146	0.328	
	*P* value	0.061	0.021	0.0017			
*R. flavefaciens*	CD	6.64^ax^	4.22^dz^	5.82^aby^	1.720	< 0.0001	< 0.0001
	KH	6.60^ax^	6.54^cx^	6.05^ay^	0.301	0.0022	
	LL	5.74^by^	7.16^ax^	5.27^cz^	0.980	0.0008	
	KHLL	5.88^by^	6.85^bx^	5.48^bcy^	0.706	0.0094	
	*P* value	0.0005	< 0.0001	0.0311			
*Fibrobacter succinogenes*	CD	5.61	5.21	4.96	0.324	0.1410	0.13
	KH	5.89^x^	5.19^xy^	5.02^y^	0.459	0.0712	
	LL	5.28^xy^	5.69^x^	4.84^by^	0.429	0.0238	
	KHLL	5.88	5.71	5.63	0.129	0.8660	
	*P* value	0.082	0.485	0.112			

CD (control diet), KH (*Kleinhovia hospita* diet), LL (*Leucaena leucocephala* diet), KHLL (*Kleinhovia hospita* and *Leucaena leucocephala* mixed diet), SEM (standard error of mean), DXT (diet time interaction for all the treatments)

^abcd^Means with different superscripts along the same column are significantly different (*P* < 0.05)

^wxyz^Means with different superscripts along the row are significantly different (*P* < 0.05)

#### Protozoa

The total protozoa population in the rumen of goats was not influenced by dietary treatments at 0 and 12 h postprandial. However, at 4 h postprandial, the control goats had greater (*P* < 0.05) population of total protozoa compared with those fed tree forages. The presence of tannin in the tree forages could be responsible for the decrease in the population of protozoa. This observation is consistent with the finding of Singh et al. (2011) who reported a reduction in the rumen protozoa count in cows and buffalos fed pakar leaves compared to oat leaves. In contrast, Benchaar et al. (2008) did not observe significant differences in protozoa numbers in dairy cattle fed quebracho tannins (CT concentrations of 700 g/kg, 150 g/day).

#### Methanogens archaea

Goats fed tree forages had lower (*P* < 0.05) population of methanogens at 4 and 12 h postprandial. This observation could be attributed to the reduced population of protozoa in the goats caused by the presence of tannin in the diets. Similarly, tannin reduced the population of methanogens (Kim et al. 2015). Contrary to the present observation, supplementation of pakar leaves increased the population of methanogens in goats (Singh et al. 2011).

#### Cellulolytic bacteria

Dietary supplementation of tree forages influenced the populations of *R. albus* and *R. flavefaciens* but did not affect the population of *Fibrobacter succinogenes* in the rumen of goats. Goats fed LL, KH, and KHLL diets had a greater (*P* < 0.05) population of *R. albus* than the control goats at 4 and 12 h postprandial. The response of *R. flavefaciens* to dietary tree forages was inconsistent. The increase in the population of *R. albus* in the rumen of goats supplemented with tree forages could be due to the reduced population of protozoa or the high-fiber content of the diets. Similarly, high forage diet increased the population of cellulolytic bacteria in beef cattle (Carberry et al. 2012).

## Conclusion

The results of the present study showed that the supplementation *K. hospita*, *L. leucocephala*, and their mix could be used in the diet of goats fed urea-treated rice straw without compromising rumen metabolism and microbial population.
